# Some Gastric Symptoms and Gastric Diseases

**Published:** 1911-09-30

**Authors:** William P. S. Branson

**Affiliations:** Assistant Physician Royal Free Hospital.


### Hospital Clinics.

### SOME GASTRIC SYMPTOMS AND GASTRIC DISEASES.

By WILLIAM P. S. BRANSON, M.D., M.R.C.P., Assistant Physician Royal Free Hospital.

(A Clinical Lecture delivered at the Medical Graduates' College and Polyclinic.)

I wish to bring before you to-day some points concerning gastric symptoms and gastric diseases, their inter-relations, and occasional independence. Most gastric disorders produce gastric symptoms, but not all. Conversely, most gastric symptoms depend upon gastric disorders, but a good many of them do not; and it happens not infrequently that to treat gastric symptoms as being significant of gastric disorders is not only useless but actually harmful to the patient. My subject falls, therefore, into two chapters—

1. Gastric disorders producing symptoms other than gastric;
2. Gastric symptoms due to diseases not gastric.

First, then, of gastric disorders producing symptoms elsewhere. This is a small group. Apart from the vague disturbances of health which may be the heralds of gastric carcinoma (I allude particularly to the pseudo-pernicious anæmia occasionally attending this disease), I know of two items only which properly find their place under this heading. The first is gaseous distension of the stomach, which fairly commonly produces symptoms apparently not gastric. These symptoms are:—

(*a*) Circulatory symptoms, *e.g.* faintness or cardiac irregularity.

(*b*) Dyspnœa.

Here are some examples. A schoolgirl of twelve years is subject to attacks of faintness, but never actually loses consciousness. Physical examination reveals no anomalies of note beyond a great extension of the tympanitic stomach-resonance, which reaches high up into the left axilla. The child is not robust in appearance, and somewhat anæmic. Treatment with an acid solution of arsenic and iron produces a fairly rapid improvement, and the symptoms and signs disappear in the course of a few weeks. Again, a boy of ten is brought for treatment because he seems so constantly out of breath and is continually sighing. Here, also, physical examination reveals no disease, but again merely an increase in the extent of the stomach note, and treatment upon the lines above indicated leads to steady improvement.

The second item in this group is rare, but its recognition is of the greatest moment. I allude to the remote and tardy symptoms occasionally excited by an unidentified perforation of a gastric ulcer. I propose to narrate two such cases, but can only show you one, for the other patient died. This was her history.

A woman of twenty-seven was seized one day with a sharp pain under the heart, and vomited. Four days later I saw her for the first time. She said she had not vomited since the early hours of her illness, and complained of pain in the left side of the chest. Her temperature was 103°, her respirations somewhat rapid—28 to the minute—and examination gave the physical signs of a small effusion at the base of the chest on the left side. The belly seemed natural, and the case appeared to be one of pleural effusion, probably empyema. But presently she developed an elastic tumour in the pelvis, pronounced by a gynæcologist to be a pelvic abscess, probably pyosalpinx. It turned out to be an abscess, and was evacuated per vaginam, without any noticeable amelioration of the symptoms. Fever continued high, and was accompanied by frequent rigors. On the ninth day of the illness the normal cardiac dulness had given place to an area of tympanitic resonance extending upwards as high as the third rib on the left side. The left epigastric region was the seat of a definite bulging, also tympanitic, and over the whole of the tympanitic area it was possible to elicit a well-marked bell-sound. These findings, of course, immediately illumined the whole case. It was obvious that there existed a subdiaphragmatic abscess. The initial attack of pain had signified the moment of perforation of a gastric ulcer. The resulting inflammation had spread through the diaphragm and excited the pleurisy with effusion which had dominated the clinical picture when I first saw her. Later, an abscess formed beneath the diaphragm, and, leaking, tracked downward to the pelvis, and there produced a secondary abscess. Post-mortem examination (for the patient died) showed a perforated gastric ulcer on the anterior surface of the stomach, near the cardiac orifice, and near the lesser curvature. Looking back I see that the misleading character of this illness depended primarily upon the lapse of time between its onset and my first examination. For this interval had sufficed to allow an entirely secondary lesion, the pleurisy, to become well-established and to masquerade as an adequate cause of the symptoms.

This case met me early in my career, and being somewhat dramatic, it lived in my memory. I think if I had not seen it, I should not have been put on the right track in the analogous case I have now to show you. Here, in brief, is his history. A man of thirty-eight years was admitted to the Royal Free Hospital complaining that he had been incapacitated for eleven months by pain in his left loin and in the left side of his chest.* He described the pain as paroxysmal, sharp and stabbing at its onset, fading later to a dull aching which never left him. His previous health had been good with the exception of an attack of pleurisy four years previously, or so at least he said in the first instance. He was a tall, anæmic man of average appetite, complaining much of his pain, but of little else except slight flatulence. He exhibited a pronounced lateral curvature of the spine, with its convexity to the right. This curve extended from the first dorsal spine above to the

* This case is reported in full in the *Lancet*, May 13, 1911.

second lumbar spine below. He asserted confidently that it was but lately acquired, and attributed it to the attitude of flexion towards the left which his pain had compelled him to, assume for many months. Rest and dieting relieved him temporarily, but a recurrence of the symptoms presently led to a more detailed investigation of the onset of his illness. He now admitted that though his urgent symptoms had lasted for eleven months only, he had not been really well since the attack of pleurisy four years previously. Questioned as to this attack he said that one day while at work he was seized quite suddenly by an acute pain in the left side. He went home, and the next day visited a hospital into which he was admitted, being told that he was suffering from pleurisy. His chest was punctured, and the pleurisy was pronounced to be "dry." This history of course threw suspicion upon the stomach as the primary source of all the symptoms, a suspicion confirmed by the result of cytological examination of the contents of the fasting stomach. This examination revealed an abundance of pus-cells. A singular feature of the case lay in the fact that each paroxysm of pain was accompanied by profuse sweating of the left arm and hand and of the left side of the chest, the right side remaining perfectly dry. It seemed now pretty clear that the whole illness depended upon perforation of the stomach, and in consequence an exploratory laparotomy was undertaken by Mr. T. P. Legg. It was found that almost the whole of the stomach was densely adherent to the tissues in the neighbourhood. Its anterior surface was firmly united to the anterior abdominal wall, a dense cicatrix penetrating the latter to a considerable depth. Owing to the extent of the adhesion separation could not be effected, neither was it possible to perform gastro-enterostomy, so the belly was closed without anything remedial having been effected, or so it was thought at the time. But, contrary to expectation, the operation proved a great success. Three months after it the patient expressed himself as free from pain, and had gained three stones in weight. The true reading of the case appears to be this. An unsuspected gastric ulcer gave way and excited a subdiaphragmatic abscess, with secondary pleurisy. The adhesions which bound the stomach to the anterior abdominal wall involved the terminal branches of some of the intercostal nerves, and thus gave rise to the paroxysmal attacks of neuralgic pain with the attendant reflex sympathetic phenomena, *i.e.* the sweating. Either the abdominal incision or the attempt made to separate the anterior wall of the stomach from the anterior parietes led to division of the irritated nerve-terminations, and thus brought the attacks of pain to an end.

My second chapter is concerned with gastric symptoms which do not depend upon gastric diseases. I exclude, of course, the many constitutional conditions which may give rise to vomiting, yet do not raise the suspicion of primary gastric mischief, and am concerned only with those which are liable to lead to diagnostic errors.

I must first allude briefly to *tuberculous meningitis* in early life. It happens occasionally that persistent vomiting is the first and, for some days the only, symptom of the onset of tuberculous meningitis. It is important to recognise this possibility, since it is easy to treat the symptom cavalierly as being a manifestation of some trivial digestive upset, in which case the event is difficult to explain away.

The *gastric crises of tabes dorsalis* form another group of instances in which an error of diagnosis is not infrequent, with the occasional result that gastro-enterostomy is uselessly carried out. In some of these cases there may be nothing to bring home the diagnosis unless the truth is suspected, for the clinical picture of tabes may be atypical, and ataxia absent. Here is an example. This man is forty-five years old. He said when I first saw him that he had been subject to attacks of vomiting for two years, the attacks occurring, as a rule, shortly after the taking of food. He has never had hæmatemesis, melæna, or coffee-ground vomiting. There is some epigastric pain during the attacks, but it is not a prominent feature. He is thin and haggard: his pupils are small, fixed, and equal: the knee-jerks are absent: there is no ataxia. He gives a history of syphilis some fifteen to twenty years ago.

*Adhesions.*—From time to time one meets with cases of intractable but not particularly severe epigastric pain which ordinary methods fail to relieve. It is generally easier in these instances to recommend an exploratory laparotomy than to make a diagnosis, yet with care a diagnosis can generally be made, or at least approached very closely. Here is such a case. A man aged thirty-six came under my notice on account of epigastric pain of 1½ years' duration. It was made worse by food and by physical effort, of which he undertook a great deal, being a coal-heaver. He was a man of magnificent physique and exhibited no physical evidences of disease at all, with one exception, and that a vague one. Pressure over the cæcum gave rise to the epigastric pain of which he complained, as did pressure in the epigastrium, though to a less extent. There was no evidence to incriminate either his appendix or his gall-bladder. In view of the fact that rest in bed dismissed the pain entirely, and that the epigastric pain could be excited by pressure upon the cæcum, it seemed likely that the cause of the mischief was an adhesion between the colon and the liver. For assuming this to be the case pressure upon the cæcum would exert traction upon the adhesion and thus explain the epigastric pain produced by this procedure. Exploratory operation showed that the diagnosis was nearly correct. The adhesion was not between the colon and the liver, but between the latter and the upturned free border of the great omentum. The cause of the adhesion was not discovered, but division of it relieved the symptoms.

*Gastric neurosis.*—The next, and last, patient in this series illustrates the point that occasions arise when the treatment of gastric symptoms, as though they depended upon gastric disorders, may be not only useless but harmful. This man is thirty-four years old. He came to the Royal Free

Hospital complaining of persistent vomiting of twelve months' duration, and a sense of epigastric oppression after food. For ten months he had been treated in an infirmary on the supposition that he suffered from gastric ulcer. He was decently nourished, but emotional. His knee-jerks were much increased, but there were no evidences of organic mischief of any kind. It was soon pretty evident that the foundation of his illness was a neurasthenic state, with dyspeptic symptoms which had been made inveterate by the treatment directed towards them. From the day of his admission to the hospital he never had a gastric symptom and made a good recovery as a result of efforts directed to reassuring and encouraging him.

The moral of these examples is that gastric symptoms and gastric diseases require for their proper investigation and treatment not only technical acumen, but some insight into human nature. If there is undoubted evidence of an organic lesion, though of an obscure nature, help may often be obtained by conducting a minute inquiry into the very earliest symptoms of the illness. When a doubt exists as to the presence of an organic explanation of the symptoms, I consider that the state of the knee-jerks offers a most valuable indication. For, in the absence of evidence of organic nervous disease an excessively active knee-jerk implies an emotional state, and when it exists the practitioner will do well to take the hint that the emotional condition of the patient must be attended to if success is to be achieved.

